# Projected changes to drought characteristics in Tehran under CMIP6 SSP-RCP climate change scenarios

**DOI:** 10.1016/j.heliyon.2025.e41811

**Published:** 2025-01-09

**Authors:** Mohammad Reza Khazaei

**Affiliations:** Department of Civil Engineering, Payame Noor University, Tehran, Iran

**Keywords:** Climate change, Drought, SPI, SPEI, M-LARS-WG

## Abstract

Drought represents one of the most devastating natural hazards, significantly impacting economies, societies, and the environment. Climate change is expected to alter future drought characteristics and may increase the severity of droughts. To mitigate these effects, it is essential to identify the characteristics of future droughts influenced by climate change using appropriate methods. This study aims to assess the climate change impacts on the frequency, duration, and magnitude of droughts in Tehran, the capital of Iran, which has a high concentration of the country's population and industrial activities and is currently facing water stress. Using the Modified Long Ashton Research Station Weather Generator (M-LARS-WG), capable of reproducing inter-annual variability, future projections from four GCMs under four SSP-RCP scenarios from the latest CMIP6 were downscaled. The impacts of climate change on droughts were then assessed using the SPEI and SPI drought indices. The findings suggest that M-LARS-WG was capable of accurately reproducing historical drought characteristics and performed significantly better than LARS-WG. Based on the SPEI, the duration, magnitude, and frequency of future droughts are expected to increase significantly across nearly all GCM projections. Therefore, developing proactive drought risk frameworks and mitigation strategies is essential for reducing damages from future droughts.

## Highlights

• SPEI describes drought conditions in Tehran better than SPI.
• M-LARS-WG, able to reproduce LFV, is used to assess climate change impact on drought.
• Droughts magnitude, duration, and frequency are expected to increase in Tehran.

## 1 Introduction

Drought is the most devastating natural hazard. It has significant impacts on economies, societies, and the environment. Consequently, many affected countries devote significant attention to this phenomenon in order to reduce its impacts and associated vulnerabilities [1]. There is strong evidence that droughts have become more frequent over the last century as a result of climate change driven by global warming [[1], [2], [3]]. As a result of further increases in temperature in the future, climate patterns will continue to change, and the frequency and severity of droughts are expected to increase in many regions of the world [[1], [2], [3], [4]]. Understanding the characteristics of future droughts in the context of climate change is essential for the development of effective adaptation and mitigation strategies [5]. Therefore, to effectively prepare for and mitigate the adverse impacts of droughts, it is essential to identify the characteristics of future droughts influenced by climate change through appropriate methods [2,6,7].

Typically, drought occurs due to a prolonged shortage of precipitation, often lasting for a season or longer [1,6]. Various types of droughts have been defined, including meteorological drought, agricultural drought (defined as a shortage of soil moisture), hydrological drought (defined as a shortage of streamflow), and others [6]. Accordingly, a variety of drought indices have been created to describe drought conditions. Among them, the Standardized Precipitation Index (SPI), introduced by McKee et al. (1993), and the Standardized Precipitation Evapotranspiration Index (SPEI), developed by Vicente-Serrano et al. (2010), are two of the most widely utilized indices globally [8]. Each type of drought can be associated with a specific duration based on the SPI and the SPEI [2,9]. The SPI transforms the total precipitation accumulated over a specified period, typically ranging from 1 to 24 months, into a standardized normal distribution [9,10]. This index has been utilized in numerous studies to evaluate the impacts of climate change on drought [1,2,[11], [12], [13], [14], [15]]. The SPEI transforms the difference between precipitation (P) and potential evapotranspiration (PET) accumulated over a specified period (representing the climatic water balance) into a standardized normal distribution. Considering the increase in temperature in future climates, evapotranspiration will rise, making the SPEI a suitable index for assessing the impacts of climate change on drought [2,4]. This index has been employed in various studies to assess the effects of climate change on drought [2,4,7,14,[16], [17], [18]].

Assessment of climate change impacts on droughts is generally based on general circulation model (GCM) projections. GCMs are the most effective tools for projecting future climate changes [17], as they simulate future climate series under emission scenarios. However, GCMs have a coarse resolution, requiring that their outputs be downscaled for regional climate change impact assessment [19,20]. Given the challenging and complex nature of accurately downscaling GCM outputs, a broad range of downscaling methods has been developed. Weather Generators (WGs) are among the key tools used for downscaling to assess the regional impacts of climate change [[21], [22], [23], [24], [25]].

WGs are stochastic models designed to simulate synthetic time series of various weather variables (e.g., precipitation, maximum temperature (T_max_), and minimum temperature (T_min_)) for arbitrary lengths. The parameters of WGs are derived from observed weather data to generate synthetic historical time series. These parameters are then adjusted based on GCM climate change scenarios to generate future downscaled time series [24,26]. These models offer significant advantages for assessing regional climate change impacts, including: 1) These models simulate long-term series of weather variables that have similar statistical characteristics to the baseline series but differ in details, encompassing various climate states, which is essential for risk assessment studies [27,28]. 2) WGs effectively transfer the changes in various weather statistics simulated by GCMs to the downscaled series [22,29]. 3) These models preserve the natural correlation of variables during the downscaling process, which is essential for many studies, such as assessing climate change impacts on hydrological variables or the SPEI, both of which depend on multiple variables [22,29]. Consequently, WGs have been employed in several studies to assess the impacts of climate change on droughts [26,[30], [31], [32], [33]].

Despite the mentioned advantages of WGs, these models typically underestimate inter-annual variability, including monthly standard deviations [25,29,34]. Due to this inefficiency, the distribution of monthly values in the time series of meteorological variables, and consequently, drought indices, which are typically calculated over periods of one month or more (up to 24 months), may not be accurately simulated.

The aim of this study is to assess the impacts of climate change on drought characteristics in Tehran, the capital of Iran, which has a population of nearly nine million. Climate change, population growth, and increased water exploitation have contributed to a severe water crisis in Tehran [35,36]. To achieve this objective, outputs from four GCMs under four SSP-RCP (Shared Socioeconomic Pathway–Representative Concentration Pathway) scenarios from the latest CMIP6 (a total of 16 GCM projections) were downscaled using the M-LARS-WG model, developed by Khazaei et al. (2020) [37]. M-LARS-WG, a modified version of LARS-WG, offers enhanced capabilities compared to standard daily weather generators commonly used in similar studies. Specifically, it can accurately reproduce inter-annual variability (low-frequency variability (LFV)) in weather series [37], a crucial feature for analyzing drought frequency over monthly or longer timescales. This study represents the first use of M-LARS-WG in a drought analysis. The model's performance in simulating drought characteristics based on SPI and SPEI is evaluated and compared with that of the LARS-WG model, which has been utilized in several studies to assess the impacts of climate change on these indices [[30], [31], [32], [33]].

## 2 Methodology

**Fig. 1:**
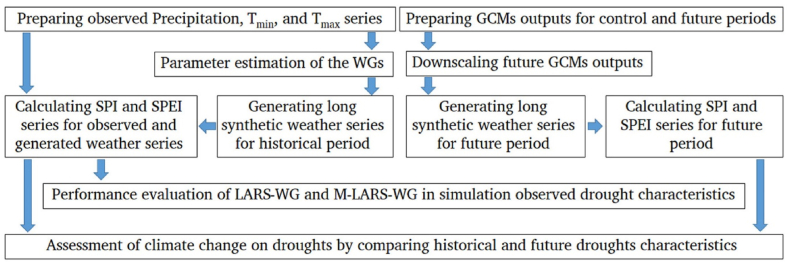
Flowchart of the research methodology.

The overall steps of the methodology are presented in Fig. 1, and the details of the methods are provided in the following sections.

### 2.1 Study region and data

The recorded daily precipitation, T_max_, and T_min_ series at the Tehran Mehrabad synoptic weather station for the period of 1984–2013 were obtained from the Iran Meteorological Organization. Table 1 presents the characteristics of the Tehran Mehrabad synoptic weather station.

**Table 1:** The characteristics of the Tehran Mehrabad weather station.

Stations	Lat. (°N)	Long. (°E)	Alt. (m)	Ecoregion division	Average Tmax (°C)	Average Tmin (°C)	Average Rainfall (mm/year)	Historical period
Tehran Mehrabad	35.68	51.32	1191	Arid	23.1	13.2	241	1984–2013

Moreover, daily precipitation, T_max_, and T_min_ data for both the control period (1984–2013) and the future period (2025–2054) from four GCM outputs under four SSP-RCP scenarios from the latest CMIP6 (16 GCM projections) were used to account for uncertainties associated with GCM structure and emission scenarios. The GCMs include the CanESM5, IPSL-CM6A-LR, MIROC6, and MRI-ESM2-0 (Table 2). The SSP-RCP scenarios include SSP119, SSP370, SSP460, and SSP585 (available at https://cds.climate.copernicus.eu/), which cover a wide range of possible future conditions.

**Table 2:** GCMs utilized in the study. All GCM simulations are based on the SSP-RCP emission scenarios from IPCC AR6.

Model	Centre	Resolution (lon. × lat.)	Control period	Future period
CanESM5	Canadian Centre for Climate Modelling and Analysis, Canada	2.8125° × 2.7893°	1984–2013	2025–2054
IPSL-CM6A-LR	Institute Pierre-Simon Laplace, France	2.5° × 1.2676°	1984–2013	2025–2054
MIROC6	A Japanese modeling community (including JAMSTEC, AORI, and NIES)	1.4063° × 1.4004°	1984–2013	2025–2054
MRI-ESM2-0	Meteorological Research Institute, Japan	1.1250° × 1.1213°	1984–2013	2025–2054

SSP119 is associated with minimal challenges in both climate change adaptation and mitigation and is linked to a radiative forcing of 1.9 W/m^2^ by 2100, resulting in low radiative forcing. SSP119 focuses on limiting global warming to below 1.5 °C, which aligns with the goal of the Paris Agreement. SSP460 involves significant climate adaptation but has low challenges for mitigation. It is linked to a radiative forcing of 6.0 W/m^2^ by 2100, corresponding to an intermediate level of future emissions and warming. SSP370 represents a scenario with challenges in both climate change adaptation and mitigation, with radiative forcing increasing to 7.0 W/m^2^ by 2100, placing it at the medium-to-high end of future emissions and warming. SSP585 is characterized by low challenges for adaptation but high challenges for mitigation, with extensive use of fossil fuels. In this scenario, radiative forcing increases to 8.5 W/m^2^ by 2100, representing the highest levels of radiative forcing.

### 2.2 M-LARS-WG

The M-LARS-WG model generates data by first creating daily series of precipitation, maximum temperature (T_max_), and minimum temperature (T_min_) using the LARS-WG model. Following this, LFV of these synthetic series is adjusted with the monthly components of the M-LARS-WG model.

The LARS-WG model [38] is a stochastic weather generator that simulates daily synthetic data for precipitation, T_max_, T_min_, and solar radiation at a specified location. This model has been widely utilized for downscaling climate model projections to evaluate the local impacts of climate change. It employs semi-empirical probability distributions to estimate the distributions of weather variables and generate corresponding data. The parameters for each weather variable's distribution are established monthly based on observed weather data series.

In the initial phase of data generation, the occurrence of rainfall is modeled by alternating between wet and dry spells, with the durations of these periods estimated using semi-empirical probability distributions tailored to each month. The amount of precipitation on wet days is then modeled by randomly sampling from the specific semi-empirical distributions for each month. To simulate the T_max_ and T_min_ series, the cycles of daily means and standard deviations are derived from the observed series based on whether the day is classified as wet or dry. Autocorrelation and cross-correlation between T_max_ and T_min_ are modeled using a first-order multivariate autoregressive model (AR(1)), which is applied to the normalized residuals. T_max_ and T_min_ for dry and wet days are estimated using semi-empirical distributions. Additional details on the LARS-WG method can be found in (Semenov and Stratonovitch (2010).

In the subsequent step, the LFV of the daily synthetic series for precipitation, T_max_, and T_min_ produced by LARS-WG is corrected using the monthly component of the M-LARS-WG model. To correct the LFV bias of the precipitation series, the frequency distribution of the monthly precipitation derived from the daily data generated by the LARS-WG model is modified to align with the frequency distribution of observed monthly precipitation via the quantile perturbation method. For this purpose, the generated precipitation series is divided into segments of the same length as the observed series, and LFV is corrected for each of them. Initially, the probability distribution of monthly precipitation for both observed and synthetic data is calculated for each month of the year. Then, throughout the synthetic daily series, the daily precipitation values for each month are multiplied by a correction factor to ensure that the total precipitation for that month matches the observed monthly precipitation corresponding to that probability. As a result, the statistical properties of the generated monthly precipitation are aligned with those of the observed monthly precipitation, and the LFV of the precipitation is adjusted accordingly.

To correct the LFV bias in the daily temperature series, a tri-variate monthly AR(1) model is applied to the observed T_max_, T_min_, and fourth root of the precipitation series. This AR(1) model generates monthly T_max_ and T_min_ series based on the monthly precipitation series obtained in the previous step. Then, the monthly mean series of T_max_ and T_min_ produced by the LARS-WG model are adjusted to align with the series generated by the monthly AR(1) model. In this way, the statistical characteristics of the daily series are aligned with the statistical characteristics of the monthly series generated by the monthly AR model, and the LFV is corrected. Additional information regarding the M-LARS-WG model can be found in Khazaei et al. (2020b).

### 2.3 Downscaling

The steps for downscaling GCM outputs using M-LARS-WG are as follows.

1 The downscaled Tmax and Tmin means are calculated using Equation (1):

(1) $$S_{i}^{Fut}={S_{i}^{Obs}+S}_{i,GCM}^{Fut}-S_{i,GCM}^{Con}$$

2 Other weather statistics used to calibrate the WGs (including daily and monthly temperature standard deviations and daily precipitation statistics) are downscaled using Equation (2):

(2) $$S_{i}^{Fut}={S_{i}^{Obs}\times S}_{i,GCM}^{Fut}/S_{i,GCM}^{Con}$$

where $S_{i}^{Fut}$ represents the future downscaled statistic, $S_{i}^{Obs}$ represents the observed statistic, and $S_{i,GCM}^{Fut}$ and $S_{i,GCM}^{Con}$ are the corresponding statistics from the GCM output for the future and control periods, respectively.

3 The quantile perturbation downscaling method is applied to transform the observed monthly precipitation distribution for each month of the year into the projected future monthly precipitation distribution.
4 Future downscaled daily T_max_, T_min_, and precipitation are generated using M-LARS-WG, based on the downscaled statistics and the projected future monthly precipitation distribution (as opposed to the observed data).

Subsequently, the SPI and SPEI series for each future scenario are calculated using the downscaled series. In calculating the future SPI and SPEI series, the parameters of the corresponding distributions derived from the historical P and D series (D = P - PET) are utilized [7].

### 2.4 Drought indices

The Standardized Precipitation Index (SPI) [9] is calculated using a monthly precipitation series. Three averaging periods of 1, 3, and 12 months were selected to represent three distinct time scales. For each observed time series, separately for each month of the year, the data is fitted to a Gamma distribution to estimate the probability values. The parameters of the distributions, hereafter referred to as 'observed series parameters,' are estimated using the maximum likelihood estimation method. The probability for any given precipitation value is determined from the corresponding gamma distribution (Equation (3))

(3) $$G\left(P\right)=\frac{1}{\beta^{\alpha }\Gamma \left(\alpha \right)}\int_{0}^{P}x^{\alpha -1}e^{-\frac{x}{\beta }}\left(dx\right)$$

where *α* and *β* are the distribution parameters, and ***Γ*** is the gamma function. This probability is then adjusted by accounting for the number of zero occurrences, as outlined in Equation (4) [16,17].

(4) $$H\left(P\right)=p_{0}+\left(1-p_{0}\right)G\left(P\right)$$

where *p*_*0*_ is the probability of the zero precipitation value. Subsequently, the corresponding value of the obtained probability is determined from the standard normal distribution, which represents the SPI corresponding to the precipitation (Equation (5)).

(5) $$SPI=F^{-1}\left[H\left(P\right)\right]$$

where *F* represents the standard normal distribution function.

The Standardized Precipitation Evapotranspiration Index (SPEI), introduced by Ref. [39], is calculated following a similar procedure to that of the SPI, with some key differences. Unlike the SPI calculation, which uses precipitation (P), the SPEI is based on the D series, representing the difference between precipitation and PET, providing a simple measure of the climatic water balance. Then, the D data is fitted to a three-parameter log-logistic distribution to obtain the probability for any given D value (Equation (6)). The log-logistic distribution, as proposed by Vicente-Serrano et al. (2010), the developers of SPEI, has been commonly used for standardizing the D series to calculate the SPEI across different time scales globally [39]. Recent studies have also adopted this distribution to standardize the D series [40].

(6) $$H\left(D\right)={\left[1+{\left(\frac{D-\theta }{\rho }\right)}^{-\omega }\right]}^{-1}$$

where *ρ*, *θ*, and *ω* are the distribution parameters. Subsequently, the corresponding value of the obtained probability is determined from the standard normal distribution, which represents the SPEI corresponding to the D value (Equation (7)).

(7) $$SPEI=F^{-1}\left[H\left(D\right)\right]$$

where *F* denotes the standard normal distribution function.

Following Vicente-Serrano (2010), who developed the SPEI, the Thornthwaite method [41] was used to generate PET. This method is advantageous as it requires only monthly mean temperature data, making it suitable for several studies assessing the impact of climate change on the SPEI [17,18,31].

As the SPI and the SPEI (according to Equations (5), (7))) follow a standard normal distribution, drought intensity for these indices is categorized as follows: −1.5 < SPI (or SPEI) ≤ −1.0 indicates moderate drought, −2.0 < SPI (or SPEI) ≤ −1.5 indicates severe drought, and SPI (or SPEI) ≤ −2.0 indicates extreme drought. A custom MATLAB code was developed by the author to calculate SPI and SPEI.

For a monthly time series of a drought index (the SPI or the SPEI for a specific time scale), a drought event and its characteristics are defined as follows: A drought event occurs when the drought index remains consistently negative and falls to −1.0 or lower. The duration of the drought event is the time between the point at which the drought index first drops below zero and when it returns to a positive value. The magnitude of a drought event is the absolute cumulative sum of the drought index values over the entire duration of the event.

### 2.5 Validation

In evaluating the performance of M-LARS-WG in simulating SPI and SPEI series, the characteristics of the generated series were compared with those of the observed data. For validation, the parameters of the distributions fitted to the P and D values are estimated based on the observed data and used to calculate drought indices for both the observed and generated series. A *t*-test was conducted to compare the means of the simulated and observed variables, an f-test was employed to compare their standard deviations, and the Kolmogorov-Smirnov (K-S) test was applied to compare the frequency distributions. These tests were performed on seasonal and annual SPI and SPEI values, as well as on drought duration and magnitude, using monthly series of 3-month and 12-month SPI and SPEI values. Additionally, the performance of M-LARS-WG was compared with that of LARS-WG.

## 3 Results

The LARS-WG and M-LARS-WG models were calibrated using observed precipitation, T_max_, and T_min_ series. Subsequently, 100 30-years of weather data were generated using each WG. Drought indices were then calculated based on both observed and generated weather data. Table 3 presents the P-values for comparing the statistical characteristics of the observed and simulated drought indices produced by the LARS-WG and M-LARS-WG models for both seasonal and annual series. P-values greater than 0.05 indicate acceptable model performance at the 5 % significance level. The P-values from the t, F, and K-S tests demonstrate that the M-LARS-WG model successfully reproduces the means, variances, and frequency distributions of the SPI series. The LARS-WG model also generally simulates the means, variances, and distributions of the SPI series acceptably at the 5 % significance level, except the variance of the spring SPI. However, the P-values for M-LARS-WG are generally larger than those for LARS-WG, indicating that M-LARS-WG performs better in simulating the SPI.

**Table 3:** P-values for the F-test, *t*-test, and Kolmogorov-Smirnov test comparing observed and simulated drought indices series at seasonal and annual time scales.

Drought Index	test	M-LARS-WG					LARS-WG				
		Winter	Spring	Summer	Autumn	Annual	Winter	Spring	Summer	Autumn	Annual
SPI	*t*-test	0.99	0.62	0.70	0.81	0.97	0.36	0.50	0.05	0.81	0.71
	f-test	0.92	0.07	0.45	0.40	1.00	0.31	0.00	0.04	0.94	0.26
	K-S test	0.85	0.96	0.90	0.76	0.63	0.57	0.50	0.06	0.91	0.32
SPEI	*t*-test	0.93	0.87	0.74	0.75	0.82	0.18	0.95	0.00	0.62	0.29
	f-test	0.34	0.37	0.79	0.68	0.43	0.05	0.00	0.00	0.17	0.00
	K-S test	0.84	0.88	0.97	0.72	0.45	0.18	0.01	0.00	0.72	0.01

The P-values for the rejected tests at the 5 % significance level (P-values <0.05) are underlined.

Additionally, the M-LARS-WG model effectively reproduces the SPEI series, with P-values consistently exceeding 0.05 across all tests. However, the P-values associated with the LARS-WG model are less than 5 % in approximately half of the tests, indicating that while its performance is nearly acceptable for generating SPEI series in winter and autumn, it is inadequate for spring, summer, and annual series.

Table 4 presents the performance evaluation results of the models in simulating drought duration and magnitude based on the monthly SPI and SPEI series at 3-month and 12-month timescales. The statistical properties (means, variances, and frequency distributions) of the simulated drought magnitude and duration are compared with those of the observed droughts. For the M-LARS-WG model, P-values from the *t*-test, F-test, and Kolmogorov-Smirnov test are all greater than 0.05, indicating that this model successfully simulates historical drought duration and magnitude based on SPI and SPEI. In contrast, the LARS-WG model did not sufficiently simulate drought magnitudes based on the 3-month SPI series at a 5 % significance level. However, its performance in simulating drought durations based on the 3-month SPI series, as well as drought durations and magnitudes based on the 12-month SPI series, is acceptable. The LARS-WG model's performance in reproducing drought statistics based on SPEI was rejected in half of the tests at a 5 % significance level. Overall, the results indicate that the M-LARS-WG model effectively simulates drought characteristics in Tehran using both SPI and SPEI indices and is therefore employed in this study to assess the impacts of climate change on drought conditions in Tehran.

**Table 4:** Performance of the models in simulating drought magnitude and duration based on monthly SPI and SPEI series at 3-month and 12-month time scales. P-values for the F-test, *t*-test, and Kolmogorov-Smirnov test are presented.

Drought index	test	M-LARS-WG				LARS-WG			
		3 Month		12 Month		3 Month		12 Month	
		Duration	Magnitude	Duration	Magnitude	Duration	Magnitude	Duration	Magnitude
SPI	*t*-test	0.06	0.17	0.56	0.46	0.12	0.00	0.41	0.79
	f-test	0.59	0.51	0.87	0.93	0.34	0.07	0.93	0.85
	K-S test	0.05	0.17	0.63	0.69	0.13	0.01	0.68	0.71
SPEI	*t*-test	0.44	0.21	0.66	0.99	0.04	0.00	0.40	0.25
	f-test	0.61	0.49	0.52	0.81	0.01	0.00	0.76	0.04
	K-S test	0.14	0.86	0.68	0.77	0.49	0.00	0.68	0.48

The P-values for the rejected tests at the 5 % significance level (P-values <0.05) are underlined.

**Fig. 2:**
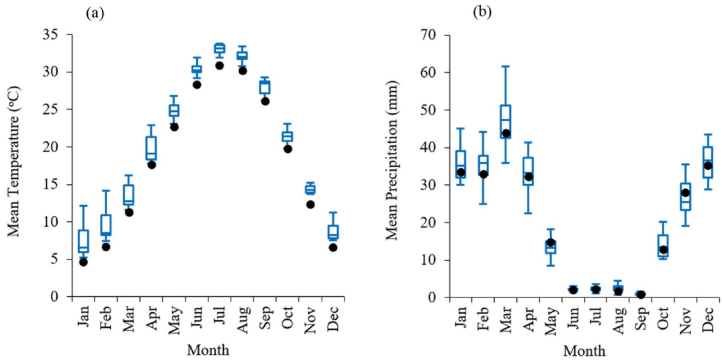
Comparison of historical monthly mean precipitation and temperature with the range of future projections from four GCMs under four SSP scenarios. Filled black circles represent historical values, and the box plot displays the median, interquartile range, minimum, and maximum values for the future period.

Fig. 2 compares historical monthly mean temperature and precipitation with the range of future downscaled values based on projections from four GCMs under four SSP scenarios (16 GCM projections). The results indicate that the monthly mean temperature in Tehran is projected to increase significantly in the future, with historical values falling outside the range of future projections. The average temperature in Tehran during the historical period was 18.1 °C. Based on the median of 16 GCM projections, the average future temperature is expected to rise to 20.1 °C, with values ranging from 19.4 °C to 21.8 °C across different scenarios (Fig. 2a). However, the average annual precipitation during the historical period was 241 mm. Based on the median of 16 GCM projections, future projections suggest an increase to 251 mm, with values ranging from 224 mm to 273 mm per year across various scenarios (Fig. 2b).

**Fig. 3:**
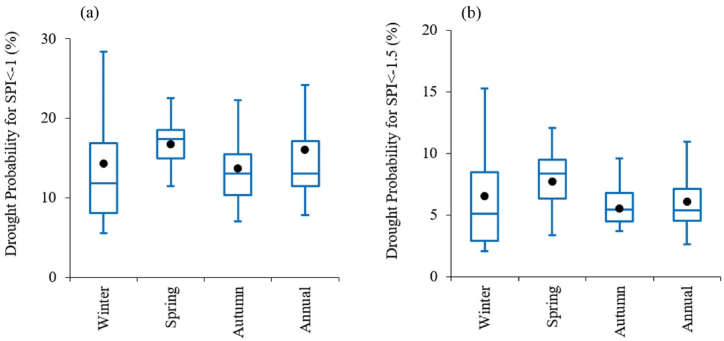
Comparison of drought occurrence probabilities between historical and future periods for (a) moderate or more intense droughts (SPI < −1) and (b) severe or more intense droughts (SPI < −1.5). Filled black circles represent historical values, and the box plot shows the median, interquartile range, minimum, and maximum values for the future period.

In Fig. 3, the drought occurrence probabilities based on SPI are compared between the historical and future periods. Overall, based on SPI, the medians of future values are close to the corresponding historical values. For example, the occurrence probability of moderate or more intense droughts (SPI < −1) based on annual data in the historical period is approximately 0.16, while in future scenarios, it ranges from 0.08 to 0.24, with a median of 0.13 (Fig. 3a). Similarly, the probability of severe or more intense droughts (SPI < −1.5) in the historical period is about 0.06, whereas, in future scenarios, it ranges from 0.03 to 0.12, with a median of 0.054 (Fig. 3b).

**Fig. 4:**
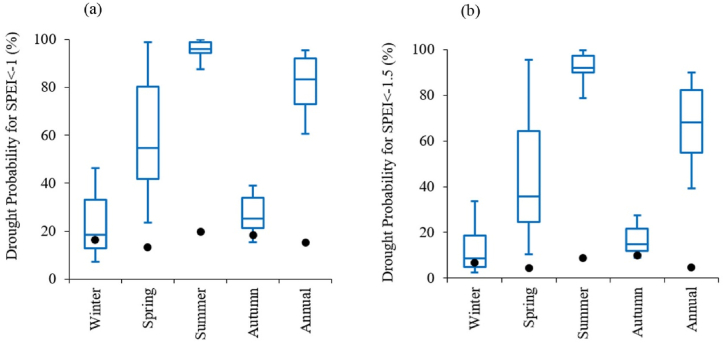
Comparison of drought occurrence probabilities between historical and future periods for (a) moderate or more intense droughts (SPEI < −1) and (b) severe or more intense droughts (SPEI < −1.5). Filled black circles represent historical values, while the box plot illustrates the median, interquartile range, and minimum and maximum values for the future period.

Fig. 4 shows the comparison of drought occurrence probabilities based on SPEI between the historical and future periods. Based on the SPEI, droughts are expected to occur more frequently in the future. For the annual time scale, the occurrence probability of moderate or more intense droughts (SPEI < −1) during the historical period is approximately 0.15, while in future scenarios, it ranges from 0.61 to 0.95 (Fig. 4a). Similarly, the probability of severe or more intense droughts (SPEI < −1.5) in the historical period is about 0.05, whereas in future scenarios, it ranges from 0.39 to 0.90 (Fig. 4b).

By comparing the median of future scenarios with the historical period, the increase in drought frequency based on SPEI, in contrast to SPI, is attributed to a significant rise in temperature, while precipitation is not expected to decrease significantly.

**Fig. 5:**
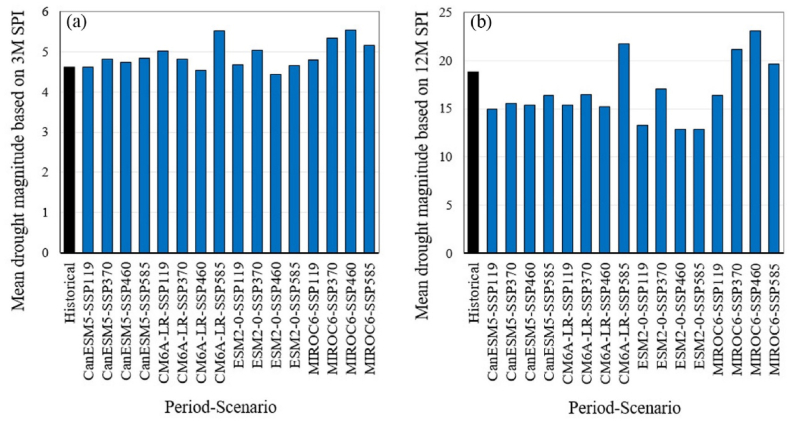
Comparison of the average droughts magnitude between the historical period and the future period based on monthly SPI series for 3-month (a) and 12-month (b) time scales. The future scenarios are based on projections from four GCMs under four different SSP scenarios.

Fig. 5 illustrates the average magnitude of droughts for the historical period and future scenarios, as assessed by the SPI series. For the monthly SPI series on a 3-month time scale (Fig. 5a), the historical value is 4.6, while future values range from 4.4 to 5.6, with a median of 4.8. The monthly SPI series on a 12-month time scale (Fig. 5b) indicates a historical value of 18.8, with future values ranging from 12.8 to 23.1 and a median of 16.

**Fig. 6:**
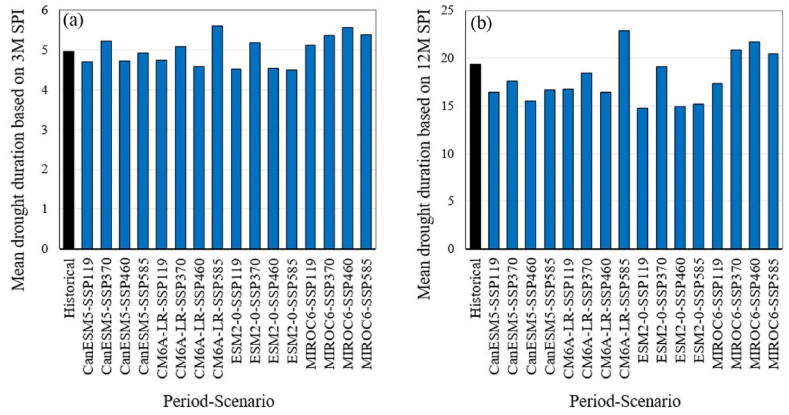
Comparison of the average duration of droughts between the historical period and the future period, based on monthly SPI series for 3-month (a) and 12-month (b) time scales. The future scenarios are based on projections from four GCMs under four different SSP scenarios.

Fig. 6 shows the average duration of droughts for the historical period and future scenarios based on the SPI series. For the monthly SPI series on a 3-month time scale (Fig. 6a), the historical average duration is 5 months, while future average durations range from 4.5 to 5.6 months, with a median of 5 months. In the case of the monthly SPI series on a 12-month time scale (Fig. 6b), the historical average duration is 19.3 months, whereas future average durations range from 14.8 to 22.9 months, with a median of 17.1 months.

**Fig. 7:**
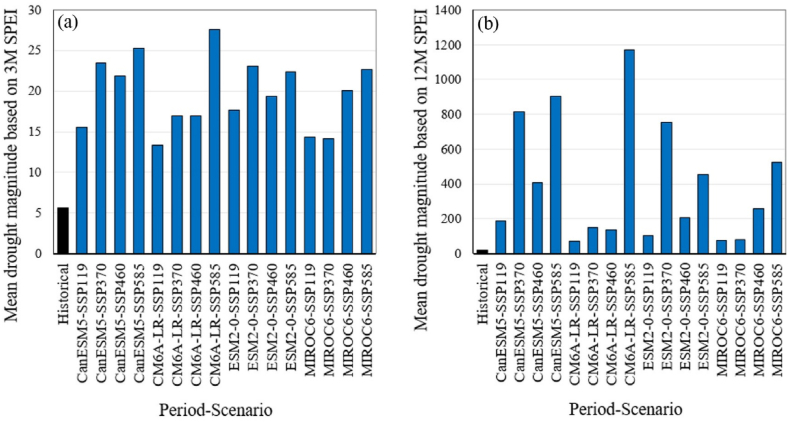
Comparison of the average droughts magnitude between the historical and future periods based on monthly SPEI series at 3-month (a) and 12-month (b) time scales. Future scenarios are based on projections from four GCMs under four different SSP scenarios.

Fig. 7 illustrates the average magnitude of droughts for the historical period and future scenarios based on the SPEI series. For the monthly SPEI series at a 3-month time scale (Fig. 7a), the historical value is 5.6, while future values are expected to increase, ranging from 13.4 to 27.6, with a median of 19.7. For the monthly SPEI series at a 12-month time scale (Fig. 7b), the historical value is 17.9, whereas future values are projected to increase significantly, ranging from 71.5 to 1173, with a median of 231.

**Fig. 8:**
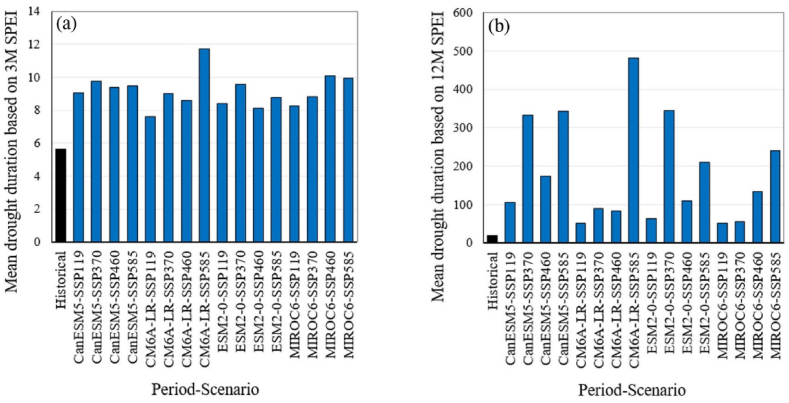
Comparison of the average duration of droughts between the historical period and the future period, based on monthly SPEI series for 3-month (a) and 12-month (b) time scales. The future scenarios are based on projections from four GCMs under four different SSP scenarios.

Fig. 8 presents the average duration of droughts for the historical period and future scenarios based on the SPEI series. For the monthly SPEI series over a 3-month time scale (Fig. 8a), the historical value is 5.6 months, while future values are expected to rise, ranging from 7.6 to 11.7 months, with a median of 9.1 months. For the monthly SPEI series on a 12-month time scale (Fig. 8b), the historical value stands at 20 months, whereas future values are projected to increase substantially, falling between 50.9 and 481 months, with a median of 121 months. The averages of drought magnitude and duration based on the monthly SPI and SPEI series at 3-month and 12-month timescales, covering both historical and future periods, are provided in Table S1 (in the Supplementary material). For the future period, Table S1 includes the median, minimum, maximum, and 25th and 75th percentiles of projections derived from the outputs of four GCMs under four SSP scenarios.

## 4 Discussion

Overall, the comparative analysis of the M-LARS-WG and LARS-WG models shows that M-LARS-WG outperforms LARS-WG in simulating drought indices and characteristics in Tehran (Table 3, Table 4). While the LARS-WG model performs adequately in simulating most SPI statistics, its limitations are evident in simulating drought statistics derived from SPEI series in about half of the cases. These shortcomings reduce the effectiveness of the LARS-WG for comprehensive drought assessments in Tehran. In contrast, the M-LARS-WG consistently outperforms LARS-WG, delivering acceptable results across all statistical tests for reproducing the statistics of SPI and SPEI series, as well as drought duration and magnitude. The superior performance of the M-LARS-WG can be attributed to its inclusion of components that account for inter-annual variability in both precipitation and temperature series. This highlights the importance of accurate modeling for future drought assessments in Tehran.

The projections highlight a significant rise in temperature and a relatively slight change in precipitation (Fig. 2). This increase in temperature is likely to result in higher evapotranspiration rates, which may exacerbate water scarcity risks despite the marginal rise in precipitation.

While projections based on SPI indicate relatively stable conditions or modest changes in drought frequency (Fig. 3), SPEI-based projections show a dramatic increase in the frequency of moderate and more intense droughts (Fig. 4). The analysis of drought magnitude and duration also reveals critical differences between SPI and SPEI projections. SPEI-based estimates predict substantial increases in both the average magnitude and duration of droughts across both 3 and 12 month timescales in Tehran (Fig. 7, Fig. 8). Conversely, SPI-based projections suggest relatively stable conditions or moderate changes in the mean magnitude and duration of droughts (Fig. 5, Fig. 6). The results are consistent with the observed trends in temperature and precipitation over the historical period in Tehran. In this period, a significant increasing trend in temperature is evident, while no significant trend is observed in precipitation [36]. The results are also consistent with the projected changes in temperature and precipitation (Fig. 2).

Rising temperature due to climate change in the future is expected to increase evapotranspiration. The increase in evapotranspiration is expected to reduce soil moisture and subsequently lead to various types of droughts, including agricultural and hydrological droughts [39]. Negative trends in the streamflow at several hydrometric stations around Tehran have been observed, as also reported in some previous studies [42]. A declining trend in annual streamflow of many Iranian rivers has also been observed in recent decades, significantly exceeding the decrease in annual precipitation [42]. This discrepancy may be attributed to rising evapotranspiration caused by increasing temperature.

The differing results based on the SPI and SPEI in this study indicate that the SPEI, which accounts for changes in both precipitation and evapotranspiration, can provide a more reliable representation of climate change impacts on droughts compared to the SPI. The results indicate that precipitation alone may not fully capture the severity of future droughts. The consistency of historical and future SPI-based drought characteristics implies that regions relying solely on precipitation-based metrics may underestimate the severity of future drought risks. These findings align with broader evidence suggesting that warming exacerbates drought conditions even in regions with unchanged or slightly reduced precipitation [3]. The results of this study in future projections are aligned with those of other studies in Iran [5].

This also underscores the importance of using a multivariate WG (e.g., M-LARS-WG) for downscaling the outputs of GCMs, which downscale temperature and precipitation while preserving their natural cross-correlation. Preserving the cross-correlation between temperature and precipitation in the generated series is essential for accurate simulation of SPEI.

The variability observed across future scenarios, particularly for SPEI, highlights the importance of using multiple GCM outputs and SSP-RCP scenarios in climate change impact assessments on droughts in Tehran. The uncertainty associated with these projections is significant, and it remains unclear which projection is most likely to occur. Utilizing a variety of GCMs and SSP-RCP scenarios captures a broader range of potential future outcomes, helping decision-makers adopt more reliable adaptive strategies and make better-informed decisions.

The results emphasize the need for preventive measures to mitigate drought impacts in Tehran, as projections based on the SPEI indicate a significant increase in the frequency, magnitude, and duration of future droughts. Key strategies may include optimal water resource management, early prediction and warning systems, sustainable water policies (e.g., groundwater protection and flood management), developing drought-resistant agriculture, strengthening water infrastructure, and enhancing social resilience and adaptation.

## 5 Conclusions

The aim of this article is to project the characteristics of future droughts in Tehran under the impacts of climate change. Tehran is a significant region in Iran and is currently facing water stress. Therefore, the unforeseen increase in future droughts could have considerable impacts in this area. To implement measures to mitigate drought-related impacts, it is essential to reliably project the characteristics of droughts under the impacts of climate change.

This paper introduces the use of a modified version of LARS-WG (M-LARS-WG) to assess the impacts of climate change on droughts. As a daily weather generator, M-LARS-WG has several advantages, such as the ability to produce long weather series, transform various changes projected by GCMs into downscaled series, and preserve the cross-correlation of weather variables during downscaling. Furthermore, it accurately reproduces inter-annual variability, which is essential for analyzing drought frequency over monthly or longer timescales, a feature not commonly found in other WGs. The results of the validation tests indicated that M-LARS-WG performed effectively in simulating various characteristics of the observed droughts, demonstrating significantly better performance than LARS-WG.

Daily precipitation and temperature projections from four GCMs under four SSP-RCP scenarios were downscaled using the M-LARS-WG. A total of 3000 years (100 30-year series) of daily data were generated for both the historical period and each future scenario. Monthly SPI series for 3-month and 12-month time scales were calculated for each dataset. The frequency of seasonal and annual droughts, along with the average magnitude and duration of droughts based on the SPI series, were compared between the historical and future periods.

The findings suggest that while temperature increases are expected to drive higher evapotranspiration rates, future precipitation trends may not mitigate the intensifying drought conditions. Based on SPEI projections, drought frequency, magnitude, and duration are expected to increase significantly under nearly all future scenarios, primarily due to rising evapotranspiration associated with increasing temperature. In contrast, projections based on SPI indicate a decrease in drought frequency, duration, and magnitude in more than half of the scenarios. The differing results between SPI and SPEI suggest that SPEI, which considers both precipitation and evapotranspiration changes, offers a more reliable representation of climate change impacts on droughts. For the monthly SPEI series on a 12-month time scale, the average duration of historical drought periods is 20 months, while future projections indicate a substantial increase, ranging from 50.9 to 481 months, with a median of 121 months. Similarly, the average magnitude of historical drought periods is 17.9, with future values expected to rise significantly, ranging from 71.5 to 1173, resulting in a median of 231. Additionally, for the annual time scale, the probability of experiencing moderate or more intense droughts during the historical period is approximately 0.15, whereas future scenarios project a range of 0.61–0.95.

This study concludes that future droughts in Tehran are likely to become more frequent and intense. Therefore, it is essential to develop proactive strategies for managing and mitigating drought risks, as well as preparing appropriate adaptation measures to effectively address these challenges in future drought risk management planning. Furthermore, with the increasing temperatures associated with climate change, warming can exacerbate drought conditions, even in regions where precipitation levels remain stable or slightly increase. As a result, projections based solely on precipitation-based indices, such as SPI, may underestimate the true severity of future droughts. Additionally, it is recommended to evaluate the performance and use of multivariate WGs capable of reproducing inter-annual variability, such as M-LARS-WG, to assess the impact of climate change on future droughts in other regions. Given the significant uncertainty associated with GCMs and SSP-RCP scenarios, considering a variety of future scenarios is advisable to obtain more reliable results.

## Data availability statement

The datasets generated or analyzed in this study are available from the corresponding author upon reasonable request. Recorded data can be obtained from the Iran Meteorological Organization, and GCM data are accessible at https://cera-www.dkrz.de/.

## Declaration of generative AI and AI-assisted technologies in the writing process

During the preparation of this work the author used ChatGPT in order to improve the readability and language of the manuscript. After using this tool, the author reviewed and edited the content as needed and takes full responsibility for the content of the published article.

## Funding

The authors received no funds, grants, or other support during the preparation of this manuscript.

## Declaration of competing interest

The authors declare that they have no known competing financial interests or personal relationships that could have appeared to influence the work reported in this paper.
