# Supplementary material for: Projected changes to drought characteristics in Tehran under CMIP6 SSP-RCP climate change scenarios
Source: Heliyon. 2025 Jan 9;11(2):e41811. doi: 10.1016/j.heliyon.2025.e41811 (PMC11774936; doi:10.1016/j.heliyon.2025.e41811)
Supplement: Multimedia component 1 [file mmc1.docx]

Table S1. Magnitude (M) and duration (D) of droughts based on monthly SPI and SPEI series for 3-month and 12-month time scales in both historical and future periods. For the future period, the median, minimum, maximum, and 25th and 75th percentiles of drought magnitude and duration are presented based on projections from four GCMs under four different SSP scenarios.

|  | SPI | | | | |  | SPEI | | | | |
| --- | --- | --- | --- | --- | --- | --- | --- | --- | --- | --- | --- |
|  | 3-month | |  | 12-month | |  | 3-month | |  | 12-month | |
|  | M | D |  | M | D |  | M | D |  | M | D |
| Historical | 4.6 | 5.0 |  | 18.8 | 19.3 |  | 5.6 | 5.6 |  | 17.9 | 20.0 |
| Future Maximum | 5.6 | 5.6 |  | 23.1 | 22.9 |  | 27.6 | 11.7 |  | 1173 | 481 |
| Future 75th percentile | 5.1 | 5.3 |  | 18.4 | 19.8 |  | 22.9 | 9.7 |  | 639 | 287 |
| Future Median | 4.8 | 5.0 |  | 16.0 | 17.1 |  | 19.7 | 9.1 |  | 231 | 121 |
| Future 25th percentile | 4.7 | 4.6 |  | 15.1 | 16.0 |  | 16.2 | 8.5 |  | 120 | 73.0 |
| Future Minimum | 4.4 | 4.5 |  | 12.8 | 14.8 |  | 13.4 | 7.6 |  | 71.5 | 50.9 |
